# Haptic Visual and Superimposed Digital Imaging Analysis Improves the Interrater Reliability of J-Sign Assessment in Patients with Patellofemoral Instability: A Prospective Diagnostic Study

**DOI:** 10.3390/jcm14238559

**Published:** 2025-12-02

**Authors:** Felix Zimmermann, Eric Mandelka, Jula Gierse, Paul Alfred Grützner, Sven Y. Vetter, Peter Balcarek

**Affiliations:** 1 Klinik Ludwigshafen, Department for Orthopaedics and Trauma Surgery at Heidelberg University, Ludwig-Guttmann-Str. 13, 67071 Ludwigshafen, Germany; 2 Arcus Sportklinik, 75179 Pforzheim, Germany; 3 Department of Trauma, Hand and Reconstructive Surgery, Departments and Institutes of Surgery, Saarland University, 66421 Homburg, Germany

**Keywords:** patellar dislocation, reproducibility of results, observer variation

## Abstract

**Background:** J-sign assessment is increasingly important for decision-making in patients with patellar instability. However, the low interrater reliability of the J-sign evaluation has raised concerns. The aim of this study was to investigate whether haptic visual assessment or superimposed digital imaging analysis might improve interrater reliability. **Methods:** In 51 patients with ≥ 1 patellar dislocation, J-sign grading was assessed by two experienced observers via three different methods: (i) plain visual evaluation; (ii) combined haptic visual assessment, including palpation of the medial and lateral patellar facets with the thumb and index finger during active knee joint motion; and (iii) a digital photo application tool using superimposed digital imaging analysis obtained at 90° of flexion–knee joint flexion and full extension. **Results:** For the visual assessment of the J-sign, the interrater reliability was fair, with κ = 0.39 ± 0.11 [0.18–0.6]. The interrater reliability of the haptic visual assessment and the photo application reached a good level of agreement, with κ = 0.89 ± 0.05 [0.8–0.98] and κ = 0.85 ± 0.05 [0.74–0.95], respectively. **Conclusions:** Plain visual evaluation of the J-sign revealed fair interrater reliability. The haptic visual assessment of the J-sign and the digital photo application tool yielded good interrater reliability. The results indicate that haptic visual assessment of J-sign should be implemented in daily clinical practice and used to communicate findings between and among physicians and studies.

## 1. Introduction

Lateral patellar instability (LPI) is one of the most common knee joint injuries in adolescents and young adults [1,2]. Reconstruction of the medial patellofemoral ligament (MPFL-R), which is injured in more than 90% of patients after first-time patellar dislocation [3], has been established as a cornerstone of surgical therapy for patients with recurrent LPI [4,5,6,7]. However, there is still no clear consensus regarding the indications and need for concomitant bony correction (e.g., correction of trochlear dysplasia, patellar height, etc.) in addition to MPFL-R [8,9]. The preoperative assessment of patellofemoral maltracking is relevant in this context. Imaging modalities such as 3D- or 4D-computed tomography scans [10,11] or dynamic magnetic resonance imaging [12] may be helpful in this regard. However, these techniques are associated with substantial costs, and dynamic MRI in particular is not widely available [12]. In this context, evaluation of the J-sign, which is a clinical sign of patellar maltracking during active knee joint motion, has been considered as a helpful parameter for decision-making [13,14,15,16,17]. In addition, the J-sign was identified as a prognostic risk factor for postoperative failure of isolated MPFL-R [16,18,19]. Accordingly, the preoperative assessment of the J-sign is becoming increasingly important in patients with recurrent patellar instability.

Classically, J-sign assessment is performed via plain visual evaluation, which involves observing the patient’s knee moving from flexion to full extension and grading the lateral displacement of the patella in full extension. However, the intra- and interrater reliability of this plain visual evaluation of the J-Sign is low [20,21,22,23,24]. Therefore, the aim of this study was to investigate whether palpation, in addition to plain visual assessment, and the use of superimposed digital imaging improved the previously reported low interrater reliability of J-sign assessment. The hypothesis was that both methods improve the previously reported low interrater reliability of plain visual J-sign assessment.

## 2. Materials and Methods

This study obtained approval from the local ethics committee of Rheinland-Pfalz and Baden-Württemberg (Reference no. 2023-16952-andere Forschung erstvotierend and B-F-2023-076) in accordance with the Declaration of Helsinki. Between October 2023 and January 2024, 51 consecutive patients with LPIs (≥1 patellar dislocation) were prospectively assessed by two raters (two consultant surgeons) at a sports medicine center and a level-one trauma center. The patients included in this study were those who presented to the outpatient clinic seeking further evaluation and management for recurrent patellar dislocations.

### 2.1. Inclusion Criteria

To be included, patients had to have an age ≥ 14 years and a history of LPI. The demographics of all patients were recorded via an internal clinic database. Moreover, in all patients, the J-sign was assessed using the modified quadrant method according to Zhang et al. [16,17]. The grading was as follows: grade 0: <1 quadrant of lateral patellar tracking; grade 1: ≥1 and <2 quadrants of lateral patellar tracking; grade 2: ≥2 and <3 quadrants of lateral patellar tracking; and grade 3: > 3 quadrants of lateral patellar tracking or complete patellar dislocation.

### 2.2. Exclusion Citeria

Patients aged <14 years and all patients without a history of LPI were excluded. Patients who had undergone previous surgery on the affected knee joint were also ruled out.

### 2.3. J-Sign Evaluation Methods

The J-sign was assessed consecutively by both raters during the patient’s outpatient visit. While one rater performed the assessment, the other was outside the examination room. Both raters evaluated the J-sign using the following three methods and recorded the determined J-sign grade in an Excel spreadsheet after each assessment, without informing the other rater.

The J-sign evaluation started with plain visual assessment, which was independently performed by the two raters as previously described [20,21,22,23,24]. For this purpose, patients were seated on the edge of an examination couch with their legs hanging over the side at 90° of knee joint flexion. Patients were then asked to slowly extend their knees from flexion to full extension, and the degree of patellar lateralization was recorded as described above.

**Figure 1:**
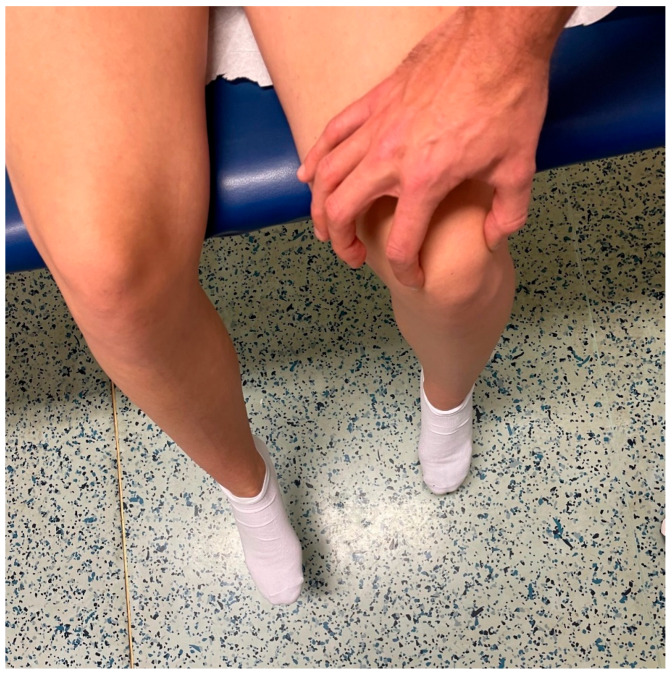
Assessment of J-sign with the haptic visual method. With the patient seated and the knee flexed at 90°, the lateral and medial patellar poles were palpated using the thumb and index finger. The patient then actively extended the knee, and patellar tracking was assessed through palpation.

This was followed by haptic visual assessment, including palpation of the medial and lateral patellar facets with the thumb and index finger during active knee joint extension (Figure 1).

Finally, J-sign assessment was performed with a newly developed digital photo application tool (“J-sign App”, mbits imaging GmbH, Heidelberg, Germany) using a superimposed picture. The digital photo application is a custom-developed, non-publicly available tool. For its development, the patellae of 20 knees from clinical staff members were marked on the skin in 90° flexion and full extension. Subsequently, photographs were taken in both positions from a perspective approximately 30 cm directly above the knee. These images were used to develop and calibrate the digital photo application.

**Figure 2:**
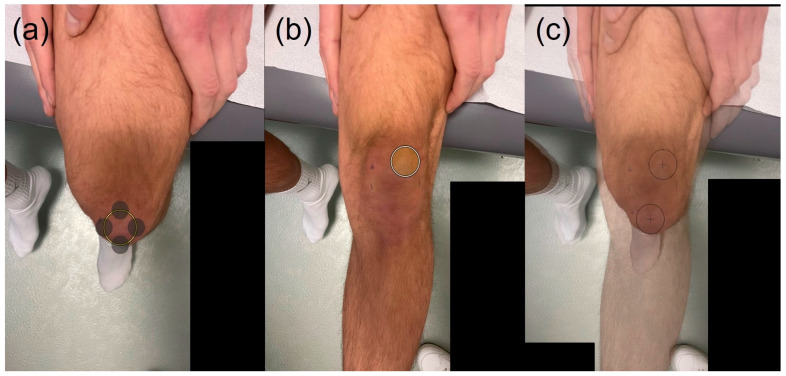
(**a**–**c**): Assessment of J-sign using the digital photo application tool. The knee was photographed at 90° flexion and full extension using a mobile app, with the patella marked by a circular template in both positions (**a**,**b**). The app calculated the J-sign grade based on the displacement between the circles using quadrant method (**c**).

For the assessment of the J-sign in patients using the digital photo application tool, a photograph of the knee joint from a perspective of approximately 30 cm in a well-lit examination room was taken at 90° of flexion using a clinic-owned mobile device equipped with the digital photo application tool (Figure 2a). Within the application, the patella was then marked with a circular template. Subsequently, a second photograph from a perspective approximately 30 cm was taken with the knee in full extension, and the patella was again marked with a circle of the same diameter (Figure 2b). The digital photo application tool subsequently calculated the J-sign grade by automatically determining the displacement between the two circles based on the quadrant method described by Zhang et al. [17] (Figure 2c).

### 2.4. Statistical Analysis

Data are presented as the mean values ± standard deviations (ranges) for numerical variables with lower and upper 95% confidence intervals in brackets. Weighted Cohen’s kappa (κ) was used to calculate the interrater reliability. All analyses were performed via GraphPad Prism (version 9; GraphPad Software, San Diego, CA, USA) and DATAtab Team (2023) (DATAtab: Online Statistics Calculator. DATAtab e.U. Graz, Austria). The level of agreement was determined according to Landis JR and Koch GG [25]. A priori power analysis was performed with G*Power (version 3.1.9.4). Using the 3 × 3 table of Bujang MA and Baharum N. [26] and the Cohen’s kappa of Hiemstra et al. [24] at the minimum level of κ = 0.31a minimum of 49 participants were calculated to achieve a power of 80% (0.8) with a given alpha error of 0.05.

## 3. Results

The study group comprised 51 patients (male/female 19/32; age 25.1 ± 10 years) with an average body mass index of 24.5 ± 5.8 (18.1–40.1) kg/m^2^ who presented for orthopedic surgical consultation for recurrent lateral patellofemoral instability.

In Table 1 the absolute and relative distributions of the J-sign grades of both raters and the three different methods used to assess the J-sign are shown

The results of the interrater reliability and the percent of agreement of the three different methods of J-sign evaluation are presented in Table 2. For the visual assessment of the J-sign, the interrater reliability was fair, with κ = 0.39 ± 0.11 [0.18–0.6]. In contrast, the interrater reliability of the haptic visual assessment and the photo application reached a good level of agreement, with κ = 0.89 ± 0.05 [0.8–0.98] and κ = 0.85 ± 0.05 [0.74–0.95], respectively.

## 4. Discussion

The most important finding of the present study was that plain visual evaluation of the J-sign showed low interrater reliability, similar to previous studies. In contrast, both the haptic visual assessment of the J-sign and the photo application yielded high interrater reliability.

In the current literature, the J-sign has been increasingly considered as important with respect to treatment decisions in patients with recurrent patellar instability. In this context, patient-reported outcome measures after isolated MPFL-R were reduced in the presence of a preoperative high-grade J-sign [16,18,19]. These findings have led to the recommendation for the correction of anatomical risk factors in addition to MPFL-R in the presence of a preoperative high-grade J-sign [13].

Traditionally, the J-sign is assessed visually, with the examiner following and assessing the extended lateral movement of the patella exclusively with their eyes. However, multiple studies have demonstrated that visual assessment alone yields limited interrater reliability [20,21,22,23,24]. Hiemstra et al. reported fair-to-moderate agreement when two raters evaluated the J-sign in patients with recurrent patellofemoral instability, whether using a binary (positive/negative) scale or a 5-point Likert scale (κ = 0.31–0.41) [24], while intrarater reliability was acceptable (κ = 0.72–0.79) in a larger cohort of clinicians using the quadrant and Donell classifications [21]. Similarly, Klimenko et al. used a quadrant-based grading system in 87 knees and found fair interobserver (κ = 0.31) and moderate intraobserver reliability (κ = 0.58) [22]. Smith et al. also reported only moderate interrater (κ = 0.53) and fair intrarater agreement (κ = 0.28) when assessing a visually defined J-sign in patients with bilateral recurrent patellar instability [23]. Using web-based assessments of standardized video recordings, Best et al. found comparable results, with moderate interrater (κ = 0.45) and intrarater reliability (κ = 0.48), and concluded that visual evaluation alone is insufficient for reliably identifying or grading patellar maltracking [20].

In contrast, Walla et al. reported substantially higher interrater and intrarater reliability (κ = 0.76 and κ = 0.75) when J-sign severity was categorized using simplified grouping systems in video assessments. Their findings suggest that methodological differences—particularly the number of grading categories—may substantially influence the reliability of visual J-sign evaluation [27].

This study aimed to overcome previous drawbacks in J-sign grading by incorporating palpation and imaging-based evaluation. The results indicate that the haptic visual assessment of the J-sign is superior to plain visual assessment in terms of interrater reliability. This multimodal perception might be associated with an improved assessment strength of lateral patellar movement. This finding is consistent with the current results on motor learning strategies. Sigrist et al. reported that multimodal feedback is more efficient than unimodal feedback [28]. Furthermore, Doyle and Snowden reported that multimodal stimuli are typically perceived more precisely and faster than unimodal stimuli are [29]. Feygin et al. showed that for learning 3D hand movements, haptic visual training of the target trajectory was more effective than unimodal training only [30]. Patrizio et al. recently published a randomized controlled trial with 32 first-year medical students who completed an educational module for cardiac auscultation. While the control group utilized traditional education methods, the interventional group incorporated multisensory stimuli with simulated cardiac cycles of 3D cross-sectioned hearts and haptic synchronization. In a post-assessment exam, the diagnostic accuracy was significantly greater in the multisensory group than in the control group. The authors concluded that the incorporation of multisensory stimuli significantly improved cardiac auscultation competency and may address the worldwide deterioration in cardiac auscultation skills [31]. These observations may explain the superiority of the haptic visual method for assessing the J-sign in terms of interrater reliability. According to the results of this study, it can be assumed that the haptic visual assessment is more reliable for communication between orthopedic surgeons and is easy to implement in daily clinical practice. The visual assessment of the J-sign can easily be adapted to the haptic visual method. In contrast to using 3D or 4D computed tomography scans and dynamic magnetic resonance imaging, it is an inexpensive and readily available option for evaluating patellar maltracking. However, the results of this study must be interpreted in light of several limitations: first, the calculation of the degree of the J-sign with the help of the photo application used in our study was based on the relative lateral displacement of the patella in two superimposed photos. However, this calculation is based on the fact that the patella must be precisely recognized on both photos and marked accordingly. This is probably the greatest weakness of this type of determination, because the exact size of the patella can often be determined only to a limited extent on the photos because of the soft tissue mantle. The reliance on manual identification of patellar landmarks inside soft tissue in the photo application introduces subjectivity that is not measured nor validated. Furthermore, the digital photo application was not calibrated during development using a more objective reference method, such as dynamic MRI imaging. Second, two raters performed the three methods for assessing the J-sign consecutively, starting with the visual method, followed by the haptic visual method and the photo application method. As a result, some bias may have been evident from the minimal washout period between the first, second and third investigations, whereby the results of the photo-application-based determination should have been the least affected. In future studies on this, the need for randomization or the use of a washout period is recommended. Third, only two raters evaluated the three methods of J-sign assessment. Moreover, the two raters were experienced in the haptic visual method. Accordingly, this could have led to the good results of this method. In contrast, both raters were unexperienced with the photo application but achieved comparable results with this method. This may lead to the conclusion that photo application may be suitable for unexperienced examiners. The influence of clinical experience on the examination is certainly not negligible. Accordingly, further studies with a larger and more diverse group of raters are necessary. Fourth, only the interrater reliability, but not the intrarater reliability, was determined for the different methods of J-sign assessment in our study. This should be performed as part of a future study. Fifth, no more objective measurements with robust reference standards, such as dynamic MRI, were available to assess the lateral displacement of the patella during knee joint movement. Finally, external validity is limited by the single center setting and the use of only two raters.

## 5. Conclusions

Plain visual evaluation of the J-sign revealed fair interrater reliability. The haptic visual assessment of the J-sign and the digital photo application tool yielded good interrater reliability. The results indicate that haptic visual assessment of J-sign should be implemented in daily clinical practice and used to communicate findings between and among physicians and studies.

## Figures and Tables

**Table 1:** Absolute and relative distributions of J-sign grades.

	Grade 0	Grade 1	Grade 2	Grade 3
Visual (Rater 1)	20 (39%)	19 (37%)	11 (22%)	1 (2%)
Visual (Rater 2)	32 (63%)	16 (31%)	2 (4%)	1 (2%)
Haptic visual (Rater 1)	17 (33%)	18 (35%)	15 (29%)	1 (2%)
Haptic visual (Rater 2)	18 (35%)	20 (39%)	11 (22%)	2 (4%)
Photo application (Rater 1)	21 (41%)	13 (25%)	15 (29%)	2 (4%)
Photo application (Rater 2)	21 (41%)	17 (33%)	13 (25%)	-

The absolute and relative distributions of the J-sign grades of both raters and the three different methods for assessing the J-sign according to the modified quadrant method of Zhang et al. [10,11] are shown. Grade 0: <1 quadrant of lateral patellar tracking; Grade 1: ≥1 and <2 quadrants; Grade 2: ≥2 and <3 quadrants; Grade 3: >3 quadrants or complete patellar dislocation.

**Table 2:** Interrater reliability of the three methods of J-sign evaluation.

	Weighted Cohen’s Kappa (κ)	Standard Deviation	Lower 95% Confidence Interval	Upper 95% Confidence Interval	Level of Agreement	Percent of Agreement
Visual	0.39	0.11	0.18	0.6	Fair	54.9%
Haptic visual	0.89	0.05	0.8	0.98	Almost perfect	90.2%
Photo Application	0.85	0.05	0.74	0.95	Almost perfect	84.3%

The values of weighted Cohen’s kappa (κ) with their standard deviations, confidence intervals and levels of agreement, as well as the percent of agreement, are shown.

## Data Availability

The datasets used and analyzed during the current study are available from the corresponding author on reasonable request.
